# The Main Patterns in the Trend Change of Stomach Cancer Incidence amongst Selected African Countries

**DOI:** 10.1155/2021/5065707

**Published:** 2021-12-23

**Authors:** Fahimeh Shokouhi, Aida Amiripour, Hadi Raeisi Shahraki

**Affiliations:** ^1^Student Research Committee, Shahrekord University of Medical Sciences, Shahrekord, Iran; ^2^Department of Epidemiology and Biostatistics, Faculty of Health, Shahrekord University of Medical Sciences, Shahrekord, Iran; ^3^Modeling in Health Research Center, Shahrekord University of Medical Sciences, Shahrekord, Iran

## Abstract

**Aim:**

The current study aimed to investigate the trend changes of stomach cancer incidence amongst African countries and identify the main patterns.

**Methods:**

The annual reports of stomach cancer incidence rate (per 100,000 people) for males and females in 53 African countries from 1990 to 2016 were maintained from the World Health Organization archive. The growth mixture model was used for fitting the models in Mplus 7.4. The estimated linear trend in each pattern was characterized by intercept (the rate at 1990) and slope (the observed biennial trend changes), and finally, each country was grouped into a cluster with the most similar pattern.

**Results:**

Three main patterns for males and two main patterns for females were determined. For males, the first cluster, containing Cape Verde, Central African Republic, and Mauritius, showed a sharp fall, while countries in the second pattern including Algeria, Côte d'Ivoire, Egypt, Gambia, Libya, Malawi, Morocco, Namibia, Nigeria, and Tunisia were categorized in a pattern with a slight decrease, and other 43 countries were in the third pattern with a moderate falling trend. For females, 19 countries including Angola, Botswana, Burundi, Cape Verde, Central African Republic, Congo Republic, Equatorial Guinea, Ethiopia, Gabon, Kenya, Mali, Mauritius, Rwanda, Sao Tome and Principe, Sudan, Swaziland, Uganda, Zambia, and Zimbabwe were categorized in the moderate-to-high falling pattern, but the other 34 countries had a gentle downward pattern.

**Conclusion:**

Although most of the observed trends of stomach cancer were falling, only a few countries had experienced a favorable decreasing trend (three countries in male incidence and nineteen countries in female incidence). Therefore, taking effective actions to accelerate the observed falling trends seems necessary.

## 1. Introduction

Africa is the most diverse and heterogeneous continent in the world. According to the World Health Organization (WHO) estimation, noncommunicable diseases have increased by 15% globally and more than 20% in Africa from 2010 to 2020. Stomach cancer is the fifth most prevalent cancer and the third leading cause of cancer death after lung and colorectal cancers worldwide [1–3]. Stomach cancer is also the 12^th^ most prevalent cancer in Africa, with a mortality and incidence rate of 3.8 and 4 per 100,000 people.

Stomach cancer incidence and mortality rates in Africa are significantly higher than in developed countries such as the United Kingdom and the United States. For example, in Mali, located in West Africa, stomach cancer is the most prevalent cancer among men, with a mortality rate of 21.1 per 100,000 and an incidence rate of 21.6 per 100,000 people [4]. Comparison of incidence rate during 1991–1993 and 1970–1980 indicates a 10-fold increase in the eastern region of Kenya. In Uganda, the incidence rate also rose from 0.8 (per 100,000) in 1960 to 5.6 in 2008, indicating a sharp increase [4]. Between 2003 and 2008, the age-standardized incidence of gastric cancer per 100,000 people was 11.1 and 11.3, respectively, among men and women in Kenya (Nairobi), 8 in Uganda (Kyadondo), 11.7 in Zimbabwe (Harare African), 2 in Malawi (Blantyre), and 5.8 in Tunisia [5]. Although the mortality rate from 1990 to 2015 for all ages and both sexes showed a slight decline, there was a considerable variation between countries [6].

Monitoring trend changes of stomach cancer and comparing different countries are known as the first steps to take potential preventive policy actions [7]. However, most previous studies were devoted to cross-sectional settings or limited to a specific country. Therefore, the lack of longitudinal studies on a large scale is tangible in this field. In this regard, this study aimed to investigate the trend changes of stomach cancer incidence among males and females in African countries.

## 2. Materials and Methods

The annual reports of stomach cancer incidence rate (per 100,000 people) in the period of 1990 to 2016 in 53 African countries were obtained for males and females from the WHO archive. These reports are freely accessible in CSV format via https://gapminder.org/data. Since annual reports for incidence rates were highly correlated, the biennial reports were considered for fitting the models in Mplus 7.4, and the Bayesian information criterion (BIC) was calculated to choose the best model with minimum error.

The growth mixture model is a powerful statistical technique to accommodate longitudinal heterogeneity of rates by clustering subjects into different latent subgroups [8, 9]. In this modeling, first, the optimum number of patterns (latent subgroups) was estimated via BIC and then the linear trend was obtained for each cluster, which is characterized by intercept (the rate at 1990) and slope (the observed biennial trend changes). Finally, each country was grouped into a cluster with the most similar pattern.

## 3. Results

**Figure 1:**
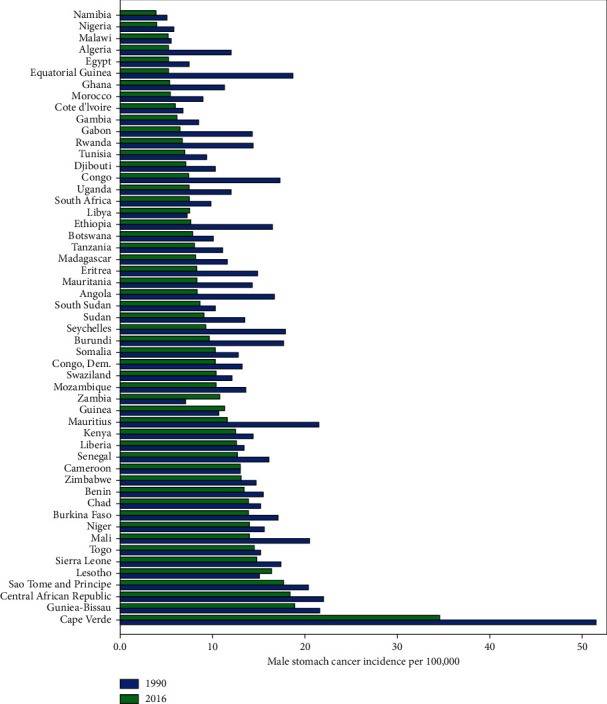
Bar chart of male stomach cancer incidence among African countries.

**Figure 2:**
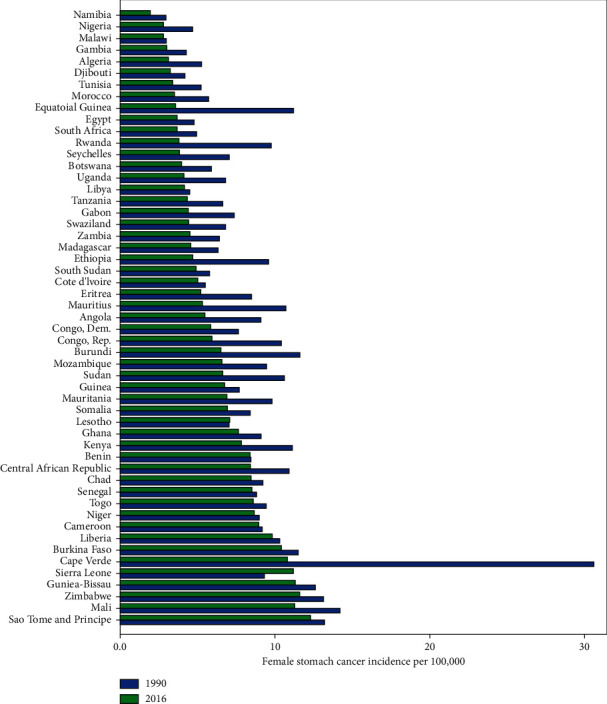
Bar chart of female stomach cancer incidence among African countries.

The longitudinal trend of stomach cancer incidence from 1990 to 2016 in 53 African countries was monitored for males and females, separately. The observed incidence for males was at the highest and lowest level, respectively, in Cape Verde and Namibia during the study period (Figure 1). Also, Cape Verde with an incidence rate of 30.6 per 100,000 had the highest rate of female stomach cancer in 1990, while in 2016, the highest rate was observed in Sao Tome and Principe with 13.3 cases per 100,000. In contrast, Namibia had the lowest rate of female stomach cancer incidence in both 1990 and 2016 years, with the rate of 2.95 and 1.95, respectively (Figure 2).

To estimate the number of patterns, results of BIC and other fit indices are summarized in Table 1, and three main patterns were determined for males and two for females. The number of countries, intercept (the rate at 1990), and slope (observed biennial trend changes) of each pattern are also presented in Table 2. For males, the first pattern including Cape Verde, Central African Republic, and Mauritius showed a sharp descending pattern while countries in the second cluster including Algeria, Côte d'Ivoire, Egypt, Gambia, Libya, Malawi, Morocco, Namibia, Nigeria, and Tunisia had a low-slope descending trend and other 43 countries were in the third pattern with a moderate falling trend.

**Figure 3:**
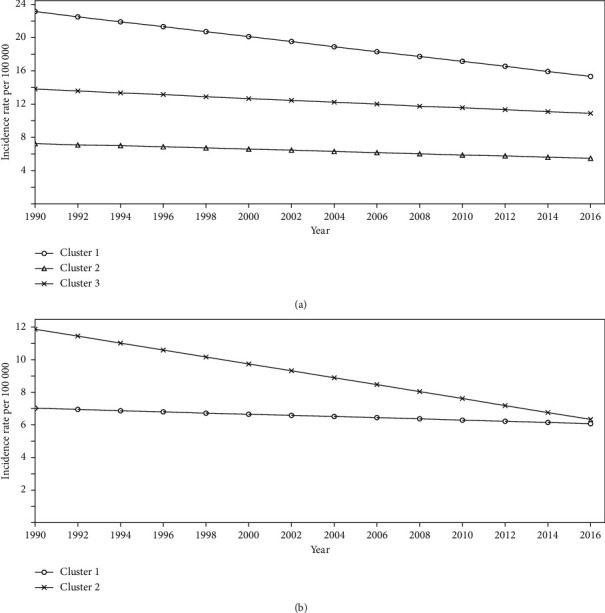
Main linear trends of stomach cancer incidence between 1990 and 2016 in Africa among males (a) and females (b).

For females, 19 countries including Angola, Botswana, Burundi, Cape Verde, Central African Republic, Congo Republic, Equatorial Guinea, Ethiopia, Gabon, Kenya, Mali, Mauritius, Rwanda, Sao Tome and Principe, Sudan, Swaziland, Uganda, Zambia, and Zimbabwe were categorized in the moderate-to-high falling pattern. On the other hand, the other 34 countries had a very low-slope downward pattern (Table 2 and Figure 3).

## 4. Discussion

The findings of this study indicate an overall declining trend in the incidence of stomach cancer in Africa. Stomach cancer is more common in developing countries than developed countries and is more prevalent among men [10]. During the last decades, the incidence of stomach cancer has decreased worldwide among both sexes [1]. The observed trends can be attributed to better identification and control of stomach cancer risk factors, such as the discovery of the role of *Helicobacter pylori* (Helicobacter) and its pathologic control [11]. Also, it may be due to the impact of dietary factors such as reducing salty and smoked and western-style foods in the diet [11], increased consumption of fruits and vegetables, and changes in environmental exposure and lifestyle in Africa [1]. However, available data regarding the cancer incidence in Africa are poor, reflecting poor diagnostic resources and lack of data collection, which could affect the reported incidence and mortality rates [4]. In many African countries, the reporting system, due to an inappropriate recording of cancer incidences, is defective, which in turn causes inaccurate estimates of the actual epidemiology [11].

The incidence of stomach cancer is relatively low in many African countries; however, the low incidence may be due to improper diagnosis of stomach cancer, and dietary differences also appear to be highly variable in different parts of Africa [12]. The most important cause of stomach cancer is Helicobacter infection, and most approaches to prevent this cancer are focused on this infection [7]. Our findings show that most of the Sub-Saharan Africa is in a cluster that has experienced a slowdown in recent decades. Consistent with these findings, Ogundipe et al. showed that between 1990 and 2015, stomach cancer mortality had decreased slightly in all ages and both sexes and in all Sub-Saharan countries [6]. In a report conducted by Mutyaba et al. in Uganda, stomach cancer was shown to decrease by 13% from 1999 to 2009, which is consistent with our findings. Also, we showed that the trend of stomach cancer incidence in Tunisia has been declining. Accordingly, in a study conducted during 1993–2006, the results showed that the APC rate among men in Tunisia was −1.3% [13].

Gastric cancer in Algeria among women aged 15–44 years decreased between 1996 and 2000 and then flattened. APC levels in men and women aged 15–44 years have been reported as −7 and 6.6%, respectively, while in all ages, it was −1.1 and 0.6%, respectively, which is in line with the present study results. This reduction in incidence is generally due to a diet rich in fruits and vegetables attributed to the improvement and preservation of nutrients and the reduction of Helicobacter infection [14, 15]. Another study from Algeria reported that the incidence was almost constant between 1986 and 2005 [16]. In another study in Algeria, which reported a five-year trend, a declined trajectory was observed with a successful decrease in Helicobacter infection. It is also probably because of changes in food storage, including less consumption of pickles and processed cigarettes and meat and more consumption of fresh vegetables and fruits [14].

In the current study, Mozambique and Sudan showed a low-slope declining or almost constant trend, which was also reported in previous studies. This trend may be due to the increasing westernization of diet, smoking, alcohol consumption, and obesity, leading to an increased risk of gastric cancer [4, 17].

One of the strengths of this study is the use of an advanced statistical method to identify the main patterns of gastric cancer incidence on a large temporal and spatial scale. One of the main limitations of this study was the small number of published studies on gastric cancer in Africa, thereby limiting their access to adequate data. Also, the reported low incidence in some areas may be because of limited diagnostic ability and insufficient statistics recording.

## 5. Conclusion

This study has tried to gain more insight into the trend of stomach cancer incidence amongst African countries to help cancer prevention by making appropriate evidence-based decisions. Although most of the observed trends of stomach cancer were falling, only a few countries had experienced a favorable decreasing trend during the last decades (three countries in male incidence and nineteen countries in female incidence). Therefore, taking effective actions to accelerate the observed falling trends seems necessary.

## Figures and Tables

**Table 1:** Fit indices for different number of clusters.

Dataset	Number of clusters	AIC	BIC	SSBIC	LRT *P* value	Entropy
Male incidence	1	2020	2057	1998	—	1.00
	2	1993	2041	1965	0.10	0.85
	3	1982	2039	1948	0.04	0.89
	4	1982	2049	1943	0.75	0.88
	5	1975	2052	1929	0.17	0.90

Female incidence	1	1571	1609	1549	—	1.00
	2	1551	1598	1523	0.35	0.94
	3	1542	1599	1508	0.26	0.92
	4	1534	1601	1494	0.28	0.95
	5	1531	1608	1486	0.49	0.91

AIC: Akaike information criterion; BIC: Bayesian information criterion; SSBIC: sample size adjusted Bayesian information criterion; LRT: likelihood ratio test.

**Table 2:** Number of countries and annual change for the identified patterns of stomach cancer in Africa.

Dataset	Cluster	Number of cancers	Intercept		Slope	
			Estimate	SE	Estimate	SE
Male incidence	1	3	23.1	8.1	−0.60	0.02
	2	10	7.2	0.2	−0.13	0.03
	3	40	13.8	0.5	−0.23	0.06

Female incidence	1	34	7.0	0.4	−0.07	0.02
	2	19	11.9	0.9	−0.43	0.02

## Data Availability

The data are freely available at Gapminder website (https://www.gapminder.org/data/).
